# Translation and cultural adaptation of the EQ-5D-Y-5L into Modern Standard Arabic for use in Egypt

**DOI:** 10.1186/s41687-025-00985-z

**Published:** 2026-02-12

**Authors:** Manar G. Elgaar, Janine Verstraete, Fatima Al Sayah, Fatma S. E. Ebeid, Maggie M. Abbassi, Samar F. Farid

**Affiliations:** 1 Department of Clinical Pharmacy, Faculty of Pharmacy, Cairo University, Cairo, Egypt; 2 Department of Paediatrics and Child Health, University of Cape Town, Cape Town, South Africa; 3 School of Public Health, University of Alberta, Edmonton, Alberta, Canada; 4 Pediatric Hematology Oncology and BMT Department, Faculty of Medicine, Ain Shams University, Cairo, Egypt

**Keywords:** EQ-5D-Y-5L, Translation, Arabic version, Health-related quality of life, Children

## Abstract

**Background:**

The use of the EQ-5D-Y-5L to evaluate children and adolescents’ health-related quality of life (HRQOL) is an essential contribution to economic evaluation methods. Accurate translation and cultural adaptation of questionnaires are crucial for their effective use, enabling comparison of data across different languages. Therefore, this study aimed to translate and culturally adapt the EQ-5D-Y-5L into Standard Arabic for use in Egypt.

**Methodology:**

The translation process was done according to the methodology and translation guidelines approved by the EuroQol Group Version Management Committee. Two forward translations into Arabic, then two back translations to English were performed, independently. The third step was conducting cognitive interviews with the children, which included a card-ranking exercise, self-completion of the translated Arabic questionnaire, and detailed discussion with the children about the questionnaire sentences. The last step was proofreading of the final Arabic version of the questionnaire.

**Results:**

The first Arabic versions of the paper and digital questionnaires were created after the forward translation. Following the back translation, only one modification was made in the digital source of the Arabic version. Cognitive interviews were conducted with 11 children with a mean age of 10.6 years. Some changes were suggested based on the initial 8 interviews in the Pain or Discomfort dimension and in the visual analogue scale (VAS) instructions. Three additional interviews were carried out to discuss these changes, and all were accepted. The final Arabic version was then developed after proofreading.

**Conclusions:**

The Standard Arabic version of the EQ-5D-Y-5L produced in this study is an acceptable version that can be used in Egypt and other Arabic-speaking countries.

**Supplementary Information:**

The online version contains supplementary material available at 10.1186/s41687-025-00985-z.

## Background

Quality of life (QOL) is a valuable outcome in research related to health and medicine, and research on quality of life involves different populations and study designs. The QOL assessment is essential for evaluating the outcomes of disease and treatment and making patient-centered medical decisions [1]. The use of health-related quality of life (HRQOL) questionnaires is an essential contribution to economic evaluation methods, including cost effectiveness and cost utility analysis [2]. Evaluating children and adolescents’ HRQOL has also become more popular, both in medical practice and research settings. As a consequence, a number of tools have been developed to assess children’s and adolescents’ HRQOL [3]. The FDA recommendations regarding instruments used with children and adolescents are to consider vocabulary specific to their age, language understanding, and grasp of the health concept assessed [4]. The most commonly used preference-based measure in this context is the EQ-5D. This preference is because of its ease of use, availability of many country-specific value sets and it is widely used in research studies [2].

The EQ-5D was developed in 1990 by the EuroQol Group as a generic tool for measuring and valuing HRQOL [5]. The EQ-5D instrument consists of a descriptive system and an EQ VAS (visual analogue scale). EQ VAS is a self-rating vertical scale of health that ranges from 0 (worst imaginable health) to 100 (best imaginable health). ‘Mobility’, ‘self-care’, ‘usual activities’. ‘pain/discomfort’. and ‘anxiety/depression’ are the five dimensions of the descriptive system. There are three levels for each dimension: (no, moderate/some, and unable to perform/extreme problems) [6]. In order to enhance the instrument’s sensitivity to small changes in health and to decrease ceiling effects, the number of severity levels in each dimension was expanded. Therefore, a version which includes five severity levels for each dimension, was developed and called the EQ-5D-5L, and the existing version was renamed the EQ-5D-3L [7].

The EQ-5D-3L/5L were standardized and originally intended for use in adults over the age of 18 as a HRQOL generic measure [6]. However, researchers started using them for younger ages. In 2010, the Youth version EQ-5D-Y-3L was developed by the EuroQol group by making modifications to the EQ-5D to be suitable for children [8]. EQ-5D-Y-3L is a self-complete, generic, and suitable for children tool that is used to measure HRQOL for ages 8 to 15 (children and adolescents). In order to make the dimensions’ meaning clear to children, some changes particularly in the dimensions wording were made, as there was agreement that the five dimensions were essential for measuring the HRQOL of children as well. Therefore, the five dimensions of the EQ-5D-Y-3L were renamed to “walking about”, “looking after myself”, “doing usual activities”, “having pain or discomfort”, and “feeling worried, sad, or unhappy” [9]. The EQ-5D-Y-5L, the officially newer version of the EQ-5D-Y-3L, with five severity levels (no, a little bit, some/quite, a lot of/really, and cannot/extreme problems) was developed for the same reasons as the adult version, and investigations of its psychometric performance are still ongoing [10].

Researchers from seven countries collaborated on the modification process to develop the EQ-5D-Y-3L (pilot version) in English and then translated it into Italian, German, Swedish, and Spanish [8]. The EQ-5D-Y-5L was developed in English, Spanish, Swedish, and German [10]. It is now available in multiple languages and different administration modes [11]. However, there is no Arabic version of the instrument. Arabic is the fifth most widely spoken language in the world, with 491 million speakers with Egypt having the largest population, about 108 million [12, 13]. The number of children and adolescents in Egypt between 10 and 14 years old is about 10 million, and between 5 and 9 years old about 12 million [14]. In order to use the EQ-5D-Y-5L in the (Modern Standard Arabic) to assess the HRQOL of Egyptian and possibly other Arab-speaking children and measure its psychometric properties, translation and cultural adaptation are needed. Therefore, this study aimed to translate and adapt the EQ-5D-Y-5L to Modern Standard Arabic language for use in Egypt.

## Methods

Since the EQ-5D-Y-5L has not been translated to Standard Arabic in any Arab-speaking country, the English version for the UK was used as the reference instrument in this translation process, in addition to the EQ-5D-Y-3L Arabic version for Egypt. The translation process was performed according to the EuroQol Group’s translation guidelines, following methodology and instructions approved by the EuroQol’s Version Management Committee (VMC) [15]. This process was performed by independent translators for forward and backward translations and three of the authors, who are native Arabic speakers, fluent in English, and have clinical pharmacy background. Ethics approval (CL 3060) was obtained from the Research Ethics Committee of the Faculty of Pharmacy at Cairo University.

**Fig. 1:**
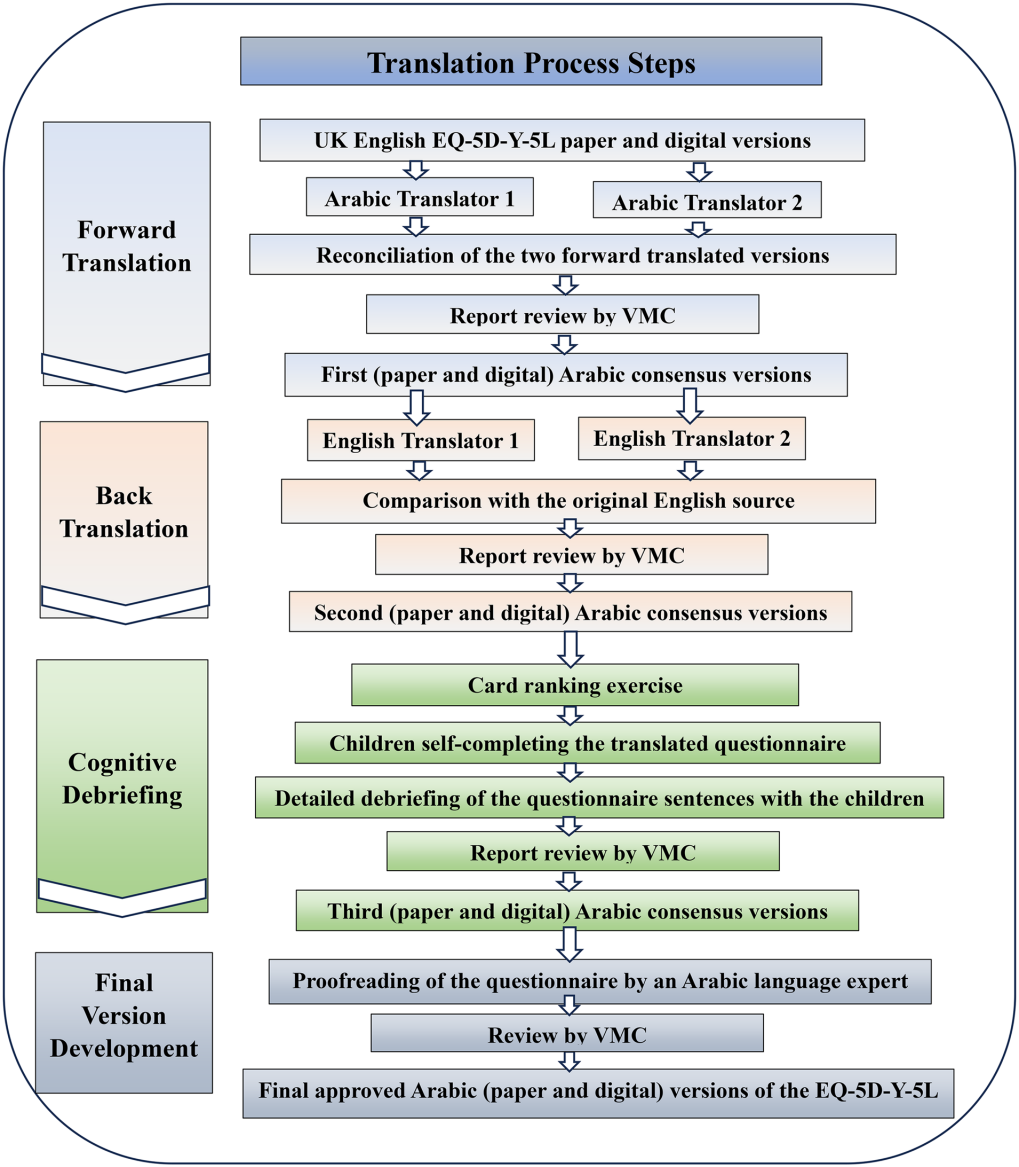
The translation process steps of the EQ-5D-Y-5L Arabic version

The general steps of translation process (Fig. 1) included:

1. Forward translation.
2. Back translation.
3. Cognitive debriefing.
4. Final version proof-reading.

### Forward translation

EuroQol’s VMC recommends conducting translations for the paper and digital versions of the EQ instruments concurrently. As such, two forward translations of the English EQ-5D-Y-5L paper and digital versions into the Arabic language were done independently by two professional translators, who are native Arabic speakers and fluent in English.

The research team then reconciled the two forward translated versions, to create the first Arabic version draft. Then, a report containing the first agreed-upon Arabic version with comments discussing controversial words, conflict points, and proposed decisions was shared with the VMC for review. After the draft consensus version had been finalized and approved by the VMC, the first (paper and digital) Arabic versions were produced.

### Back translation

The forward translated Arabic versions were back translated to English by two independent professional translators who are native English speakers and fluent in Arabic, and unfamiliar with the original English EQ-5D-Y-5L version.

The research team then compared the two translations to each other and to the original source of the questionnaire (the English for the UK version). They noted the differences between them, agreed on the changes required for the first Arabic version and sent the report on the back translation process to the VMC reviewers. Based on the feedback, the second Arabic version was developed.

### Cognitive debriefing

The wording chosen for the Arabic version was evaluated through face-to-face interviews with children to decide whether it is clear, understandable as intended, and suitable for their age or needs modifications. The cognitive interviews were performed according to the translation guidelines for cognitive debriefing by EuroQol group, which is composed of three stages: card ranking exercise, children self-completing the translated Arabic questionnaire, and finally structured detailed debriefing of the questionnaire sentences with the children [16]. We aimed to recruit 3 children aged 8–9 years, 3 children aged 10–11 years, and 2 children aged 12–15 years, with half having a chronic health condition and half of them being males [15]. We conducted 11 one-to-one interviews with Egyptian children aged 8–15 years who were able to self-complete the questionnaire and provided assent and written consent from parents. The first 8 respondents participated in all three steps of the cognitive interview, and the additional 3 respondents participated in the second and third steps only as per the recommendations of the EuroQol VMC. This was because the additional three interviews were intended to discuss new terms and certain modifications in the EQ VAS instructions that were developed after the initial eight interviews, so the VMC did not recommend including the ranking exercise in these additional interviews. All respondents were recruited by convenience sampling. All interviews were conducted in Arabic by a trained, native Arabic interviewer who is also able to communicate in English. Then, the responses of children were translated into English to be included in the report and analyzed. The interviews took place in the presence of parents, either in the participants’ homes or the interviewer’s home, and some interviews were conducted in the Faculty of Pharmacy at Cairo University. All interviews followed the same ethical and procedural standards, participant comfort, and confidentiality.

### Card ranking exercise

As the number of levels in the EQ-5D-Y-5L increases, it becomes more difficult to accurately translate severity levels. Therefore, the card ranking exercise was developed and included prior to the other steps in the cognitive interview by the VMC guidelines [16]. This was done to confirm that the hierarchical order of the levels in the translated version is clear and consistent with the severity order in the original English source before the participants saw the translated instrument which showed the correct rank of severity. The exercise involved arranging sets of cards that included the translated severity qualifiers of dimensions (no problem, a little bit of a problem, some problems/quite, a lot of problems/really, cannot/extreme problems) onto five empty boxes in a column on a sheet of paper to represent their relative severity between two points, “the biggest problem” and “the smallest problem” for the child (Appendix 1 Supplementary Material) [16].

The children ranked all sets of cards under the supervision of the interviewer, and any understanding issues raised in the introductory set were addressed to ensure that the children clearly understood the exercise before proceeding to the other four sets of the questionnaire dimensions (mobility, looking after myself, pain or discomfort, and worried, sad or unhappy). The usual activities dimension was not included, as it contains the same severity qualifiers for the mobility and looking after myself dimensions [16]. Then the interviewer recorded the order of severity levels and difficulties facing the child in each set in the data sheet.

### Self-completion and detailed debriefing of the EQ-5D-Y-5L questionnaire

After finishing the ranking exercise, the children were asked to complete the second Arabic consensus version of the EQ-5D-Y-5L questionnaire by themselves. The time to complete the questionnaire and the difficulties and any assistance given to children were recorded by the interviewer. Then the detailed interviews were done to discuss each sentence with the child, asking them about the questionnaire wording and whether there were any terms they found difficult to understand or if they could describe in their own words what they were asked to do. The children were then asked to give an example of someone who had a problem similar to the problems described in the dimension’s levels. The responses of the children were then translated to English. Some changes to the second Arabic version were suggested by the research team and the VMC reviewers based on the children’s responses. After approval, the third Arabic consensus version was produced.

The final step was proofreading of the questionnaire by a native Arabic-speaker linguist for grammatical, spelling, or layout errors. Then the final approved Arabic version of the EQ-5D-Y-5L questionnaire was produced (Appendix 2 Supplementary Material).

## Results

After obtaining ethics approval, the study was conducted between January 2023 and January 2024. The study involved a convenience sample of healthy and sick Egyptian children and adolescents aged 8 to 15 years. The children differed in socioeconomic status from low to middle status, in geographic location from Cairo and Menoufia Governorates, and in the types of schools to ensure diversity of characteristics. The total respondents in the study were 11 children (6 girls and 5 boys) with a mean age of 10.6 years, and there were 6 healthy and 5 children with chronic conditions (Table 1).

**Table 1:** Demographic characteristics of the respondents

Characteristics	*N* (%)
Participants	11
Sex	
Male Female	5 (45.5) 6 (54.5)
Age groups – years	
8–9 10–11 12–15	4 (36.4) 4 (36.4) 3 (27.2)
Health Condition	
No health problems Have a chronic condition	6 (54.5) 5 (45.5)

* Data is presented as Numbers (Percentages)

### Forward and back translation

The two forward translations were comparable to some extent; the research team revised these two translations while considering the Arabic version of the EQ-5D-Y-3L to be consistent with it as much as possible. All sentences in Arabic Y-3L that were clear and did not differ from the two translations were taken as is. During the development of the first consensus Arabic version of the Y-5L some changes were made to words in the Y-3L Arabic version to make them clearer and more understandable for this age range. In the instructions ‘Under each heading’, the word used in the Arabic Y-3L was difficult for the children to understand and was not a correct translation of ‘heading’, so we used another word that both translators agreed upon. In the walking about dimension, the second level ‘I have some problems’ was translated in the Arabic Y-3L version using the word that means ‘I suffer’ not ‘I have’, so we changed this word with an appropriate word for ‘I have’ in the Y-5L Arabic version. This change was agreed upon by the VMC reviewers, and we also suggested changing this word in the Y-3L Arabic version. In the third dimension ‘Doing usual activities’, we changed the word used in the Y-3L Arabic version as it meant ‘practice’ and used the simpler word for children which is the correct translation for ‘doing’. We aimed to be consistent in translation and selection of the severity qualifiers for the five levels across dimensions. Therefore, the severity qualifiers chosen for the levels in the first three dimensions of the Arabic Y-5L version were the same, and the words chosen were clear, appropriate for children, and able to differentiate between levels in severity. Also, severity qualifiers chosen for the levels in the fourth and fifth dimensions of the Arabic Y-5L version were the same. The words chosen for these two dimensions were not the literal meaning of the severity qualifiers of the Y-5L English version (quiet, really, and extremely) in Arabic, because they would be confusing and difficult for this age group to differentiate between them, so we chose simple words that made the difference in the severity grades between the levels more obvious. The differences between EQ-5D-Y-3L and EQ-5D-Y-5L Arabic versions are presented in Appendix 3 Supplementary Material.

In the back translation, the questionnaire was translated into an English version comparable to the original source. There were no modifications made to the first Arabic consensus version after the back translation except for one phrase from the digital source: ‘This item is blank, please complete it’. The Arabic word that was chosen to translate the word ‘item’ was back translated to ‘element’ and ‘value’ by the two translators, which didn’t imply the meaning of ‘item’, so we changed it to another Arabic word, which was then translated by both as ‘item’.

### Cognitive debriefing

#### Ranking exercise

**Fig. 2:**
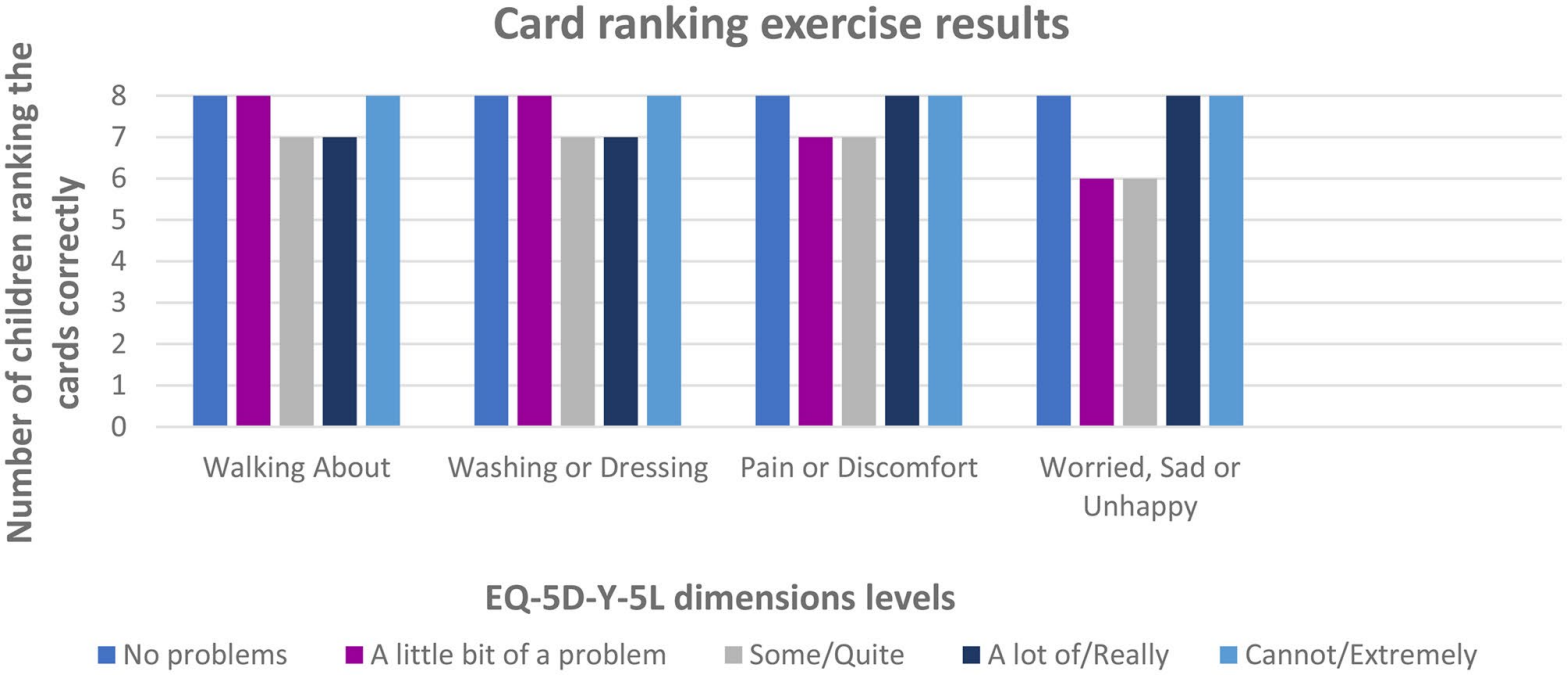
Number of children correctly ranking the cards in Ranking Exercise. *Each bar represents one of the five response levels of the EQ-5D-Y-5L questionnaire

The ranking exercise was conducted at the beginning of the interview before the children saw the questionnaire and before the detailed cognitive debriefing to ensure that they didn’t know the correct order of the dimension severity levels. Eight respondents participated in the ranking exercise; each ranked the five severity level cards across four sets of questionnaire dimensions, resulting in a total of 160 ranked cards. There were no difficulties facing children in deciding how to order the cards in all sets, Fig. 2 represents the number of children who correctly ranked the cards across dimensions. The children ranked all dimensions correctly as intended, except for 10 out of 160 rankings (6.25%) that were incorrect (Table 2). Four of these incorrect rankings were for one child, mixing between ‘some problems’ and ‘a lot of problems’ levels in the (walking about) and (washing or dressing) dimensions. The remaining incorrect rankings were between second and third levels for the (pain or discomfort) and (worried, sad, or unhappy) dimensions. These three children with incorrect rankings were a little confused during the ranking exercise, but later, as the interview went on, they had no problem and were able to give examples indicating that they understood the ranking.

**Table 2:** Ranking exercise results

EQ-5D-Y-5L dimensions	Box ranking order for Respondents (*R*)							
	R1 (8–9)	R2 (10–11)	R3 (8–9)	R4 (12–15)	R5 (8–9)	R6 (10–11)	R7 (12–15)	R8 (10–11)
Set 1 (Walking About)								
No problems	1	1	1	1	1	1	1	1
A little bit of a problem	2	2	2	2	2	2	2	2
Some problems	3	3	3	3	3	3	3	**4**
A lot of problems	4	4	4	4	4	4	4	**3**
Cannot walk about	5	5	5	5	5	5	5	5
Set 2 (Washing or Dressing)								
No problems	1	1	1	1	1	1	1	1
A little bit of a problem	2	2	2	2	2	2	2	2
Some problems	3	3	3	3	3	3	3	**4**
A lot of problems	4	4	4	4	4	4	4	**3**
Cannot wash or dress myself	5	5	5	5	5	5	5	5
Set 3 (Pain or Discomfort)								
No pain or discomfort	1	1	1	1	1	1	1	1
A little bit of	2	2	2	2	2	2	**3**	2
Some	3	3	3	3	3	3	**2**	3
A lot of	4	4	4	4	4	4	4	4
Extreme	5	5	5	5	5	5	5	5
Set 4 (Worried, Sad or Unhappy)								
Not worried, sad or unhappy	1	1	1	1	1	1	1	1
A little bit	2	**3**	2	2	2	2	**3**	2
Quite	3	**2**	3	3	3	3	**2**	3
Really	4	4	4	4	4	4	4	4
Extremely	5	5	5	5	5	5	5	5

* Numbers in bold indicate incorrect ranking

### Self-completion and detailed debriefing of the questionnaire

The summary of the cognitive debriefing results presented in Table 3. The mean (SD) time children spent completing the questionnaire was 5.2 min (2.18). All children responded that the questionnaire was clear, easy to understand and answer, and not too long except for one child, aged 9 years, who said that the questionnaire was too long even though the child completed it in 4 min. Older children, > 11 years, generally had no issue understanding the instructions, and this problem appeared mainly with younger children. However, most children were able to understand the questionnaire general and VAS instructions when they either read them for a second time or after asking the interviewer to confirm their understanding.

The children then took part in a standardized cognitive interview according to the VMC guidelines. All children were able to give examples that confirmed their understanding of the hierarchical order of levels. For example, in the ‘walking about’ dimension, an example for ‘a little bit of a problem’ level was someone with leg pain; for ‘some problems’ level it was someone with severe leg pain; for ‘a lot of problems’ it was someone with a broken leg who walked on crutches; and for ‘cannot’ level it was someone who moved in a wheelchair. In the ‘looking after myself’ dimension, examples of ‘a little bit of a problem’ and ‘some problems’ levels were ‘someone with pain in hand who can’t move it’ and ‘someone with a broken hand and leg’ respectively. In the ‘doing usual activities’ dimension, an example of ‘a little bit of a problem’ was a child with a common cold; for ‘some problems’ it was a child with a broken leg; and for ‘a lot of problems’ it was a child with an illness that would take a long time to heal or a child with cancer. The examples for the levels of the ‘worried, sad, or unhappy’ dimension were ‘feeling a little worried when going to an unfamiliar place’, ‘feeling quite sad when someone gets sick’, ‘feeling really worried and sad when going to get a blood sample for CBC’, and ‘bullying makes a child feel extremely sad and unhappy’.

In the dimension of ‘having pain or discomfort’, we found that the addition of the term ‘in my body’ to the dimension name in the translation was appropriate and important. However, some children thought of examples of psychological pain or discomfort, such as ‘a child whose father died and whose circumstances were not taken into account by the school would feel a lot of pain, and a sick child who has to go to school for exams would feel a lot of discomfort’. For this reason, the VMC reviewers discussed this with the research team and suggested using another term for pain that would refer mainly to physical not psychological pain. In addition, there was a change suggested by the children in the digital source: ‘This item is blank, please complete it’. Five of the children didn’t understand the Arabic word chosen for ‘item’, and four of them suggested replacing this word with ‘question’ and we also suggested that to be more suitable for the children.

The VMC then requested that we conduct three additional cognitive interviews that included questionnaire self-completion and detailed debriefing to discuss these new terms and some modifications in the EQ VAS instructions with the children. All children in the additional interviews were able to provide examples of physical pain at all levels; for example, someone with a headache for a little bit of pain, someone with a common cold for some pain, someone who had a car accident for a lot of pain, and someone who had heart surgery for extreme pain. Also, the new word for ‘item’ was tested in the additional interviews and was found to be understood as intended. All suggested changes were accepted based on the results of the additional interviews.

**Table 3:** Characteristics of cognitive debriefing interviews

Characteristics	*N*
Total number of interviews	11
Number of interviews involved all 3 steps: card ranking exercise, children self-completion, and detailed debriefing of the questionnaire	8
Number of interviews involved only children self-completion and detailed debriefing of the questionnaire	3
Responses about the EQ-5D-Y-5L Clear Easy to understand and answer Not too long	11 11 10
Number of respondents needing confirmation about their understanding for the general and VAS instructions of the questionnaire	6
The mean time (SD) needed to complete the questionnaire (min)	5.2 (2.18)
The mean time (SD) needed to complete all cognitive debriefing interview (min)	38 (14.5)

VAS: visual analogue scale, SD: Standard deviation, Data is presented as numbers and mean time (SD)

## Discussion

The aim of cultural adaptation is to develop semantically and linguistically equivalent versions of the questionnaire in different languages. These versions can convey the same meaning as the original version, and respondents who speak different target languages can interpret them similarly [17]. In this study, we followed a strict and comprehensive translation process following EuroQol VMC guidelines that align with internationally used guidelines by ISPOR (International Society for Pharmacoeconomics and Outcomes Research), to ensure thorough forward and back translation, cognitive debriefing, card ranking exercise, and proofreading [18]. The newly developed Arabic version of the EQ-5D-Y-5L questionnaire was found to be clear and easy to understand and was answered by all children who participated in the study. There were no problems facing older children in completing the Arabic questionnaire and understanding the instructions, but some younger children (8–11 years old) needed to read the instructions twice to be able to understand what they should do and to ask for confirmation from the interviewer that they understood well. However, they were able to complete the questionnaire on their own and give examples in the cognitive interview which showed their understanding of the order of the severity levels. Based on these findings, we recommend using the interviewer-based version for the younger children aged 8–10 years to ensure that they answer correctly.

The cognitive interviews revealed that all children were able to provide examples of the severity levels of the dimensions that reflected their understanding of their sequence. There was an issue that arose when we asked the children about the ‘having pain or discomfort’ dimension; even though the term “in my body” was added to the dimension name in the translation, some children still thought of examples of emotional rather than physical pain. For this reason, the term used for pain was changed to the new term often used with Arabic-speaking children, which refers usually to physical, not emotional pain. This problem also appeared in other translations; in the Chichewa version, they changed the term used for discomfort to another term that refers to feeling uncomfortable, and in the Singapore English version, they added examples of discomfort such as breathlessness, aches, and itching to make it easier for children to understand the term ‘discomfort’ [19, 20]. Also, in the Bahasa Indonesia Y-5L version, they used the term referring to (body) in the discomfort dimension to avoid misunderstanding the term as psychological [21].

We found that the card ranking exercise with Arabic speaking children in Egypt aligned with the study that assessed three exercises with children in Spain and New Zealand and found that the card ranking exercise was preferred by nine out of twelve children [16]. Also, in the translation of the Y-5L into Bahasa Indonesia, they used the rating exercise, but they recommended using the card ranking exercise, as it was later used to test the final version and confirm its comprehensibility for children [21]. The ranking exercise results revealed that children were able to understand and differentiate between levels of severity and that the chosen Arabic words were appropriate. Three out of eight children had inversions between severity qualifiers, and this was consistent with the results of other countries: three out of nine children in South Africa and two out of ten children in Indonesia also had inversions [16].

Although the translation and adaptation of the EQ-5D-Y-5L questionnaire into Standard Arabic provided good results, and it was the first Arabic study to our knowledge that focused on the youth 5L version, there are limitations to consider. The Arabic language is unique with complex grammatical structure and rich vocabulary, which made the translation process challenging. Some English terms may not have direct equivalents in Arabic, so translators had to pick words carefully to ensure that they convey the same meaning. There are also many regional dialects in Arabic countries that can vary significantly from each other [22]. This study used Standard Arabic, which can be used in other Arabic-speaking countries after validation in the local context and thus can serve as a reference instrument in adapting it to local cultures in other Arabic-speaking countries. The psychometric properties and performance of the newly translated Arabic version of the EQ-5D-Y-5L questionnaire have been assessed in different health conditions to promote its widespread use. The results will be published separately.

## Conclusion

The Standard Arabic version of the EQ-5D-Y-5L produced by following the EuroQol translation guidelines and cultural adaptation with Arabic-speaking children in Egypt, is an acceptable version that can be used to assess quality of life in different health conditions in Egypt and possibly many Arabic-speaking countries and allow comparison of results between different studies.

## Supplementary Information

Below is the link to the electronic supplementary material.

Supplementary Material 1

## Data Availability

The datasets generated from this study are available from the corresponding author on a reasonable request.

## Abbreviations

HRQOL: Health Related Quality of Life
QOL: Quality of life
VAS: Visual Analogue Scale
VMC: Version Management Committee
ISPOR: International Society for Pharmacoeconomics and Outcomes Research
