# Supplementary material for: Translation and cultural adaptation of the EQ-5D-Y-5L into Modern Standard Arabic for use in Egypt
Source: J Patient Rep Outcomes. 2026 Feb 12;10:35. doi: 10.1186/s41687-025-00985-z (PMC12946566; doi:10.1186/s41687-025-00985-z)
Supplement: Supplementary file 1 — Supplementary Material 1 [file 41687_2025_985_MOESM1_ESM.docx]

**Supplementary Materials:**

**Appendix 1. Card Ranking Exercise Material**


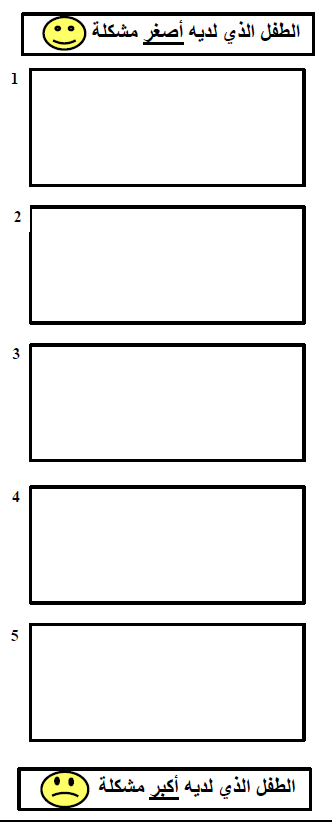

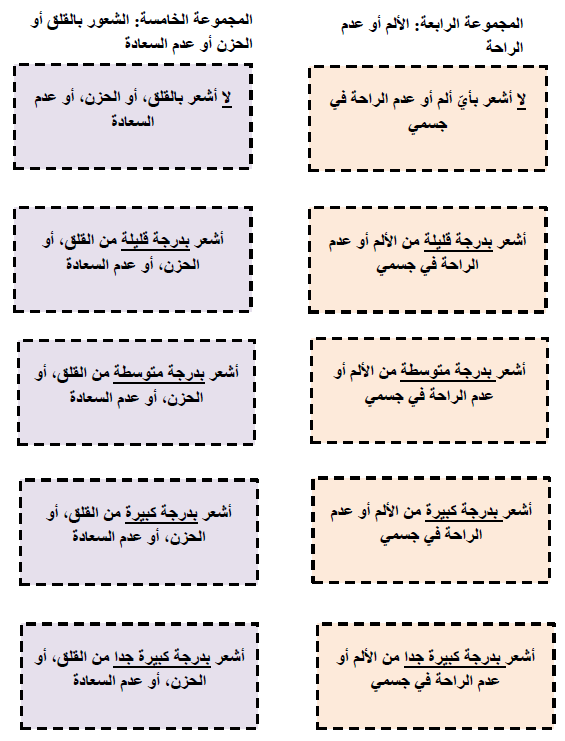


**Appendix 2. EQ-5D-Y-5L Arabic version *(This is for illustration purposes only; the EQ-5D-Y-5L must be requested directly from the EuroQol Group)***

|  | تحت كل عنوان، يُرجى وضع علامة في مربّع **واحد** فقط يصف بأفضل صورة حالتك الصحية **اليوم**. |
| --- | --- |
|  |  |
|  | القدرة على الحركة *(المشي)* |
| ❑ | ليس لديّ أيّ مشاكل أثناء المشي |
| ❑ | لديّ مشكلة بسيطة أثناء المشي |
| ❑ | لديّ بعض المشاكل أثناء المشي |
| ❑ | لديّ الكثير من المشاكل أثناء المشي |
| ❑ | لا أستطيع المشي |
|  |  |
|  | العناية بنفسي |
| ❑ | ليس لديّ أيّ مشاكل عند الاستحمام، أو ارتداء الملابس بنفسي |
| ❑ | لديّ مشكلة بسيطة عند الاستحمام، أو ارتداء الملابس بنفسي  DRAFT |
| ❑ | لديّ بعض المشاكل عند الاستحمام، أو ارتداء الملابس بنفسي |
| ❑ | لديّ الكثير من المشاكل عند الاستحمام، أو ارتداء الملابس بنفسي |
| ❑ | لا أستطيع الاستحمام، أو ارتداء الملابس بنفسي |
|  |  |
|  | الأنشطة المعتادة *(مثل: الذهاب إلى المدرسة، أو الهوايات، أو الرياضة، أو اللعب، أو القيام بأشياء مع العائلة أو الأصدقاء (* |
| ❑ | ليس لديّ أيّ مشاكل في القيام بأنشطتي المعتادة |
| ❑ | لديّ مشكلة بسيطة في القيام بأنشطتي المعتادة |
| ❑ | لديّ بعض المشاكل في القيام بأنشطتي المعتادة |
| ❑ | لديّ الكثير من المشاكل في القيام بأنشطتي المعتادة |
| ❑ | لا أستطيع القيام بأنشطتي المعتادة |
|  |  |
|  | الشعور بالوجع أو عدم الراحة في جسمي |
| ❑ | لا أشعر بأيّ وجع أو عدم الراحة في جسمي |
| ❑ | أشعر بدرجة قليلة من الوجع أو عدم الراحة في جسمي |
| ❑ | أشعر بدرجة متوسطة من الوجع أو عدم الراحة في جسمي |
| ❑ | أشعر بدرجة كبيرة من الوجع أو عدم الراحة في جسمي |
| ❑ | أشعر بدرجة كبيرة جدا من الوجع أو عدم الراحة في جسمي |
|  |  |
|  | الشعور بالقلق أو الحزن أو عدم السعادة |
| ❑ | لا أشعر بالقلق، أو الحزن، أو عدم السعادة |
| ❑ | أشعر بدرجة قليلة من القلق، أو الحزن، أو عدم السعادة |
| ❑ | أشعر بدرجة متوسطة من القلق، أو الحزن، أو عدم السعادة |
| ❑ | أشعر بدرجة كبيرة من القلق، أو الحزن، أو عدم السعادة |
| ❑ | أشعر بدرجة كبيرة جدا من القلق، أو الحزن، أو عدم السعادة |

أفضل حالة صحية

يمكن أن تتخيلها

0

5

10

15

20

25

30

35

40

45

50

55

60

65

70

75

80

85

90

95

100

| - نود أن نعرف مدى جودة أو سوء حالتك الصحية **اليوم***.* |
| --- |
| - هذا الخط مرقم من الرقم 0 حتى 100. |
| - بحيث يشير الرقم 100 إلى أفضل حالة صحية يمكن أن تتخيلها. والرقم صفر إلى أسوأ حالة صحية يمكن أن تتخيلها. |
| - يُرجى وضع علامة (×) على الخط للإشارة إلى مدى سلامة أو سوء حالتك الصحية **اليوم**.   DRAFT |
| - الآن، اكتب الرقم الذي وضعت عليه علامة في المربع بالأسفل. |

صحتك اليوم =

أسوأ حالة صحية

يمكن أن تتخيلها

**Appendix 3. The differences between EQ-5D-Y-3L and EQ-5D-Y-5L Arabic versions**

| Differences | UK English EQ-5D-Y-5L | Arabic EQ-5D-Y-3L | Arabic EQ-5D-Y-5L |
| --- | --- | --- | --- |
| Instructions | Under each heading | 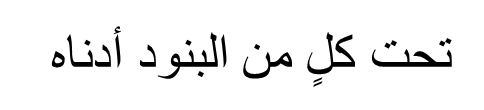 | 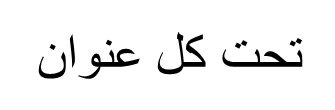 |
| Response options for Mobility (Walking about) | I have some/a lot of problems walking about | 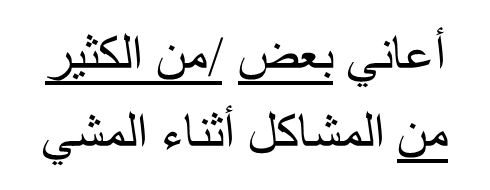 | 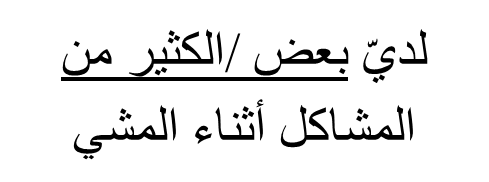 |
| Response options for Doing Usual Activities | …… doing my usual activities | 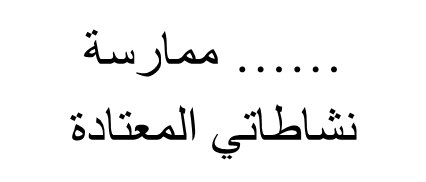 | 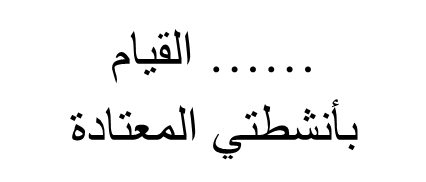 |
| Dimension name | Having Pain or Discomfort | 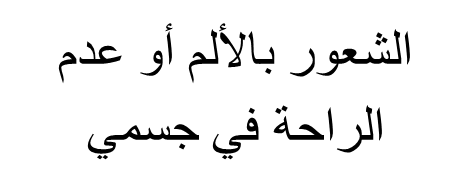 | 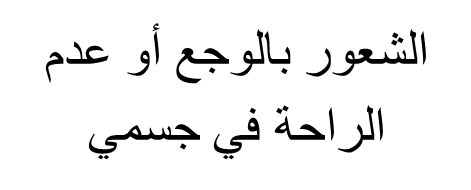 |
| Response options for Having Pain or Discomfort | I have some/a lot of pain or discomfort | 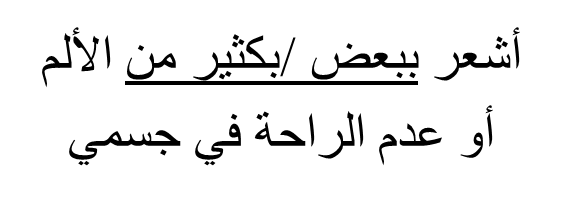 | 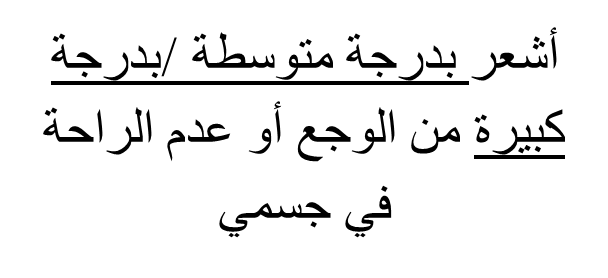 |
| EQ VAS instructions | This line is numbered from 0 to 100. | 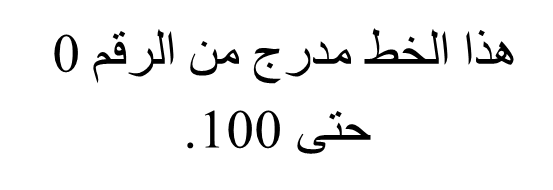 | 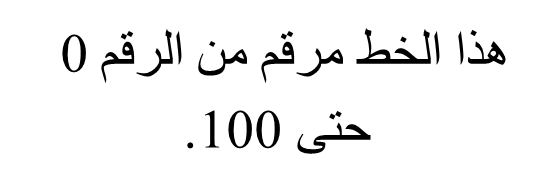 |
